# Expanding the Role of Thyroid-Stimulating Hormone in Skeletal Physiology

**DOI:** 10.3389/fendo.2017.00252

**Published:** 2017-10-03

**Authors:** Ramkumarie Baliram, Rauf Latif, Mone Zaidi, Terry F. Davies

**Affiliations:** ^1^Thyroid Research Unit, Icahn School of Medicine at Mount Sinai and the James J. Peters VA Medical Center, New York, NY, United States; ^2^The Mount Sinai Bone Program, Icahn School of Medicine at Mount Sinai, New York, NY, United States

**Keywords:** TSH-β, TSH-βv, TSH-receptor, macrophage, osteoblast, osteoclast

## Abstract

The dogma that thyroid-stimulating hormone (TSH) solely regulates the production of thyroid hormone from the thyroid gland has hampered research on its wider physiological roles. The action of pituitary TSH on the skeleton has now been well described; in particular, its action on osteoblasts and osteoclasts. It has also been recently discovered that the bone marrow microenvironment acts as an endocrine circuit with bone marrow-resident macrophages capable of producing a novel TSH-β subunit variant (TSH-βv), which may modulate skeletal physiology. Interestingly, the production of this TSH-βv is positively regulated by T3 accentuating such modulation in the presence of thyroid overactivity. Furthermore, a number of small molecule ligands acting as TSH agonists, which allosterically modulate the TSH receptor have been identified and may have similar modulatory influences on bone cells suggesting therapeutic potential. This review summarizes our current understanding of the role of TSH, TSH-β, TSH-βv, and small molecule agonists in bone physiology.

## Introduction

The skeleton has a wide range of functions, which include structural support/protection, locomotion, and mineral homeostasis. In addition, the emerging role of bone as an endocrine unit is rapidly gaining momentum because bone secretes a variety of hormones such as osteocalcin, osteoprotegerin, osteoclastogenesis inhibitory factor, sclerostin, and fibroblast growth factor 23 (1), and it has recently also been shown to be the source of a variant form of TSH-β subunit (2–4). Bone is derived from intramembranous ossification of fibrous membranes and from endochondrial ossification of hyaline cartilage during fetal development. Osteoblasts and osteoclasts, two major cell types found in bone, are derived from unique cell lineages. Osteoblasts differentiate from the mesenchymal lineage while osteoclasts are from the hematopoietic stem cell lineage. The close balance in their activity during bone remodeling between the osteoblasts-inducing bone deposition and osteoclasts-inducing bone resorption appears to be crucial for precise maturation and preservation of bone integrity. However, the bony skeleton can be structurally and functionally altered by various diseases, drugs and extra-skeletal hormones, growth factors, and cytokines as well as mechanical forces (1).

An overactive thyroid gland has long been known to be associated with significant bone loss (5). Osteoporosis is seen in many overt hyperthyroid states, most commonly Graves’ disease and toxic multinodular goiter (6–9). In addition, excessive thyroid hormone replacement therapy in postmenopausal women is known to contribute to bone loss (10). Hence, bone turnover is increased and bone mass decreased when thyroid hormone levels are high and TSH levels are low and such changes in bone can also be seen in animal models (5, 11). In these conditions under which bone is loss, TSH levels in the serum fall to insignificant concentrations, but thyroid hormones (T3 and T4) may vary from high to normal, thus arguing for a role of TSH or other TSH receptor agonists in preventing bone loss. This review highlights the role of TSH, TSH-βv, and small molecules on skeletal biology.

## The Hypothalamic–Pituitary Axis

**Figure 1:**
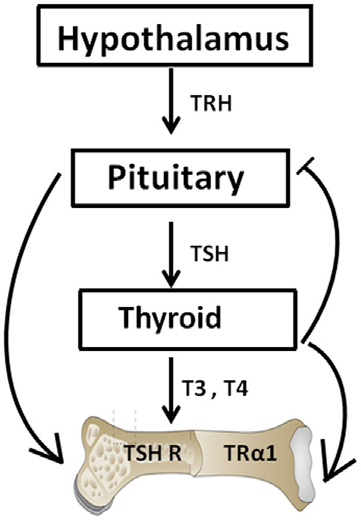
The hypothalamic–pituitary–thyroid–bone axis. This simplified figure illustrates the interactions of the pituitary hormone thyroid-stimulating hormone (TSH) and thyroid hormones T3 and T4 with bone. A negative feedback loop with origins in the thyroid and projections to the pituitary and hypothalamus is depicted. T3 and T4 hormone levels are maintained by such a loop. The role of TSH on bone has been hampered by the dogma that TSH exerts functions exclusively within the thyroid. However, within the past few decades, TSH has been show to exert physiologic effects on both osteoblasts and osteoclasts.

Thyrotropin-releasing hormone (TRH) also known as thyroliberin was first isolated by Schally and Guillemin in 1969 (12, 13). TRH is synthesized in the paraventricular nucleus of the hypothalamus and it regulates both the synthesis and release of TSH from the anterior pituitary (14). The production of the TSH-β subunit in the pituitary is regulated by both the CREB-binding transcription factors and the pituitary-specific transcription factor-1 (14) while thyroid hormone levels (T4 and T3) being preserved by a negative feedback loop (Figure 1). TSH in turn acts through the thyroid-stimulating hormone receptor (TSHR) to induce the synthesis and release of T4 and a smaller amount of T3 with additional T3 being derived by peripheral deiodination (15). Additionally, thyroid hormones then exert actions through the thyroid hormone receptors (TRs) to inhibit TRH and TSH synthesis and its secretion. As such, when thyroid hormones are high, the TSH levels are low.

## Structure and Function of the TSH Molecule

Thyroid-stimulating hormone (TSH) and follicle-stimulating hormone, along with chorionic gonadotropin (hCG) and luteinizing hormone, are heterodimeric proteins that share a common α-chain and unique β-chains, which confer hormone specificity. Both mouse and man TSH-β subunits share significant homology (16, 17). In these species, the TSH-β contains 138 amino acids with 20 of them representing the signal peptide and the other 118 the mature protein. The common α-chain is made up of 92 amino acids. The α-subunit gene shows a general expression pattern compared to the TSH-β subunit gene expression, which is restricted to the anterior pituitary. Although TSH-α and TSH-β are transcribed from different genes, it is generally understood that the molecular interaction of the α-subunit and the TSH-β subunit confers specificity to the molecule (18). TSH interacts with the G-protein-coupled TSHR (19, 20) in controlling thyroid function and it also has extrathyroidal activity *via* TSHR expression at a variety of sites (21). Of relevance here is that pituitary TSH has been shown to be osteoprotective *in vitro* and *in vivo* by activating osteoblasts and inhibiting osteoclasts and this will be reviewed further.

Mouse studies have clearly shown that there is *in vivo* osteoprotective activity associated with the TSHR itself even when pituitary TSH is suppressed by excessive thyroid hormone (11). These data indicate that either the intrinsic, constitutive, activity of the TSHR itself is able to provide the protection in the absence of TSH ligand or raised the possibility of a local TSHR stimulator being available to maintain TSHR signaling in the absence of pituitary TSH. This possibility prompted us to search for other isoform (s) of the TSH molecule in bone.

## A Novel TSH-β Subunit Variant in Pituitary and Bone Marrow

In fact, extrapituitary sources of TSH have long been known (22, 23). Hence, parallel to the pituitary-thyroid endocrine circuit, there are additional TSH-related circuits that function beyond the thyroid and involves the immune system as evidenced by reports, which shows that immune cells are capable of producing TSH (22) and a novel TSH-βv is produced within the bone marrow cells; primarily by macrophages (2–4).

**Figure 2:**
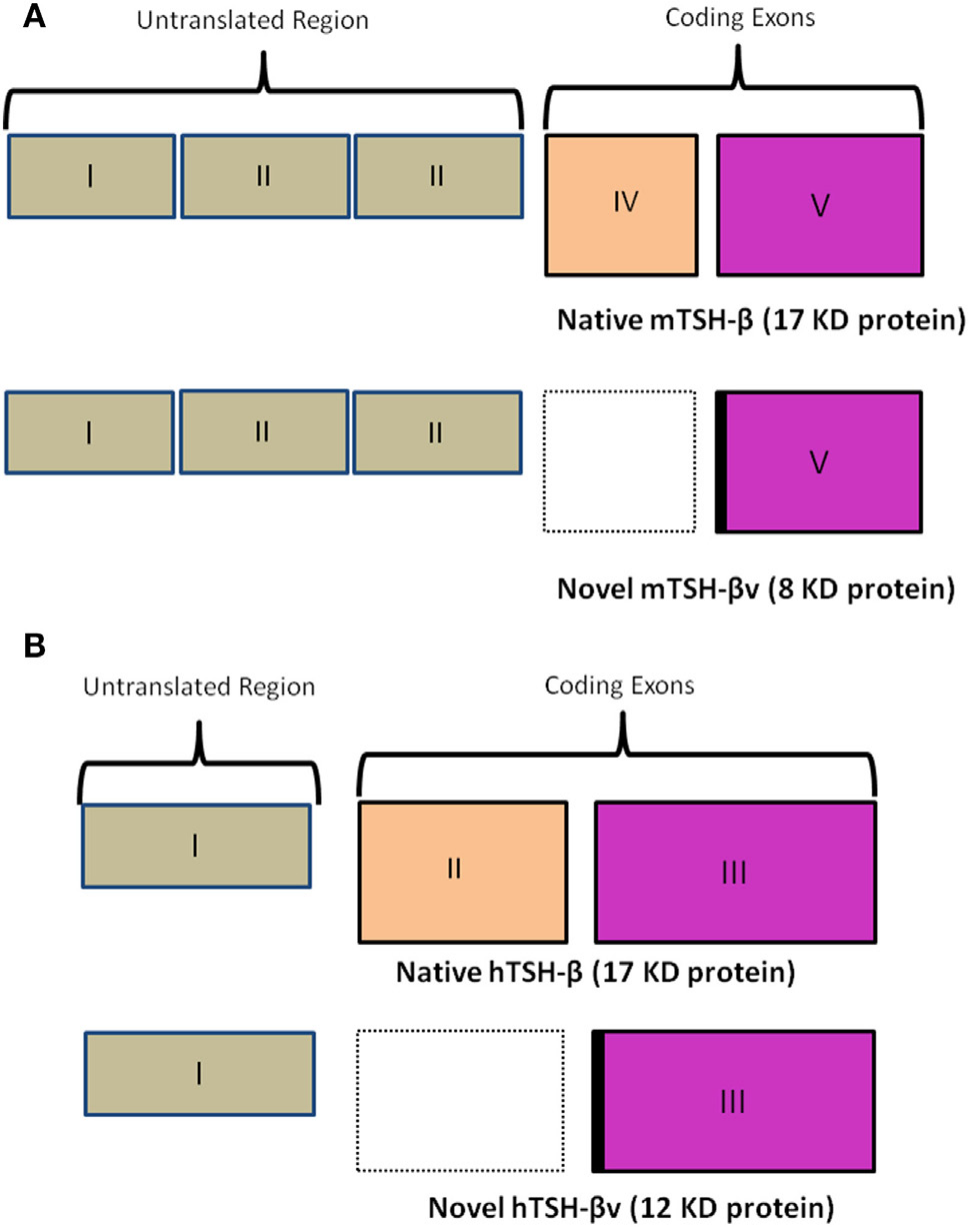
**(A)** A schematic comparison of the mouse native TSH-β and novel TSH-βv. Of note is a missing exon IV in the splice variant resulting in a smaller peptide of 8 vs 17 kDa for the full length. The intronic region is marked in black. Copyright (2013) Endocrinology and reproduced with permission from Oxford University Press (2). **(B)** A similar schematic outlining the human native TSH-β and novel TSH-βv gene arrangement [adapted from Baliram et al. (3)]. Copyright (2013) Endocrinology and reproduced with permission from Oxford University Press (2).

In the mouse (Figure 2A), unlike the human (Figure 2B), the TSH-β coding region is located in segments of exons 4 and 5. In the novel mouse, TSH-β splice variant (TSH-βv) exon 4 is missing. The human TSH gene contains three exonic sequences but exon-2 is missing in the hTSH-βv. Molecular docking and experimental studies suggested that TSH-β and TSH-βv were able to bind and signal through the TSHR (2, 3). Further, molecular docking studies have also shown that the binding affinity of TSH-βv is comparable to the native TSH-β subunit (2). Of direct relevance here is that it has been shown that the mouse pituitary in addition to macrophages is also a source of this novel TSH-β splice variant (TSH-βv), which may retain its biological effect (2–4).

In the human, TSH-β is similarly expressed primarily in the thyrotrophs of the anterior pituitary gland. But we and others have also observed, as in the mouse, that a TSH-βv is expressed in human pituitary, human bone marrow, and in human peripheral blood-derived macrophages (3, 24). These data further support the concept of an extrapituitary TSH-like molecule, which can bind to TSHRs on osteoblasts and osteoclasts to initiate proliferation and differentiation. However, the full significance of this conclusion in bone biology needs to be further elucidated.

## TSHR and Small Molecule Agonists

**Figure 3:**
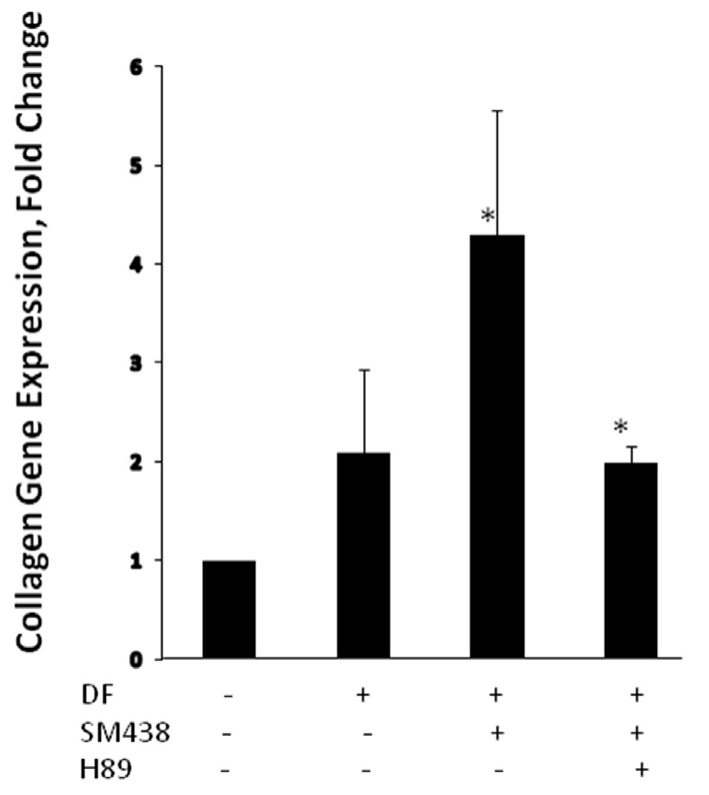
Small molecule MS 438 enhanced collagen gene expression in human osteoblast cells. The hFOB 1.19 cells were transformed into osteoblasts by treating with osteogenic stimulation/differentiation factors—20 μm of beta-glycerophosphate, 50 μg/ml of ascorbic acid, and 10^−7^M dexamethasone in refreshed media every 3 days along with or without 10 μM of MS 438 and 10 μM of PKA inhibitor (H 89). On day 10, cultures were terminated and gene expression analyzed by quantitative PCR and showed enhanced collagen gene expression.

In recent years, small molecules have gained momentum as therapeutic options for modulating TSHR signaling (25). In addition to their low cost of manufacturing, these molecules have the biological advantage of easily crossing the plasma membrane and binding to allosteric sites on the receptor. Their chemical nature renders them resistant to proteolytic enzymes and thus ideal therapeutic agents. A few potent small molecule agonists to the TSHR have been reported (26–28). These molecules interact with the TSHR on distinct polar and non-polar residues within the hydrophobic pockets created by the helices of the receptor transmembrane domains, thereby exerting a stimulatory effect by altering the interaction and movement of these helices (29, 30). Our laboratory has reported a small molecule (MS-438) (28), which appears to increase osteoblast formation through the PKA signaling pathway (Figure 3). Other studies have also shown biological action of small molecules on bone cells overexpressing the TSHR (31) and two small molecule TSHR antagonists have been reported but with lower affinity than likely to be clinically useful (27, 32).

## TSHR Gene Expression

The human TSHR gene, cloned in 1989 (19, 20), is on chromosome #14q-31 and codes for a seven transmembrane, G-protein-coupled receptor. The TSHR is the largest of the glycoprotein receptor because of its 8- and 50 amino acid insertions into the ectodomain (residues 38–45 and 317–367). The TSHR is described as a G protein-coupled receptor with both G_αs_ and G_αq_ as primary effectors and with constitutive activity, which is enhanced further by TSH or by stimulating TSHR autoantibodies (33). The minimal 5′ promoter is necessary to confer thyroid-specific expression and cAMP autoregulation (34). It is well established that in addition to the G_αs_ cAMP/protein kinase A/ERK signaling cascade, TSH activates G_αq-_AKT/protein kinase C/Ca 2+ coupled signaling networks predominantly at high concentrations (35). Although the TSHR is important for growth and function of the thyroid gland, as discussed earlier, it has a diverse expression profile including on lymphocytes, macrophages, adipose tissue, fibroblasts, heart, and bone among others (21).

## TSH Effects on Osteoblasts

Osteoblast-related cells such as bone lining cells, stromal cells, preosteoblasts osteoprogenitors, osteoblasts, and osteocytes are differentiated from mesenchymal cells. These cells can also terminally differentiate into fibroblasts, chondrocytes, myoblasts, and adipocytes (36). Osteoblast lineage cells perform varied functions, which include support for muscle attachment and lend itself as a reservoir for minerals such as phosphorus and calcium. Additionally, osteocytes derived from osteoblast lineage cells produce FGF 23 (37). Osteoblast lineage cells contribute to the bone marrow niche (38) and are also involved in insulin action (39, 40).

Osteoblast formation requires a series of sequential steps starting from precursor cell commitment, then cell proliferation and then cell differentiation, which is marked by type-1 collagen formation and matrix deposition. Once bone is formed, osteoblasts then go onto differentiate into osteocytes (41). Expression of the TSHR in the rat osteoblast line UMR106 cells was first demonstrated (42). Then in subsequent studies, the TSHR mRNA expression and protein were observed in normal osteoblasts (43–49).

Thyroid-stimulating hormone was found to induce genes involved in the regulation and differentiation of mesenchymal stem cells within the bone marrow (50) and treatment of osteoblasts with TSH *in vitro* has been shown in most studies to have stimulatory effects on osteoblast differentiation and function (31, 46, 47). Inhibition of low-density lipoprotein receptor-related protein 5 mRNA by TSH suggested a role for TSH on osteoblastogenesis. TSH has since been shown to activate Wnt-5a signaling in osteoblast differentiation (47). Similarly, in embryonic stem cell cultures, TSH-stimulated osteoblast differentiation *via* protein kinase C and the non-canonical Wnt-5a pathway (47). Further, TSH also stimulated proliferation and differentiation, as shown by an upregulation in alkaline phosphatase and in increase in IGF-1 and IGF-2 mRNA expressions (51). Recently, TSH was shown to stimulate arrestin 1, which leads to the activation of intracellular signaling molecules such as ERK, P38 MAPK, and AKT (31).

## TSH Effects on Osteoclasts

Osteoclasts are terminally differentiated polykaryons, which reabsorb bone matrix and mineral. They attach to bone through αVβ3 integrin that interacts with bone matrix proteins. These interactions form cytoplasmic extensions with finger-like processes known as the ruffled border. These borders function to increase the surface area when contacting bone and through them, osteoclasts secrete hydrochloric acid from acidic vacuoles. The acid dissolves bone mineral and also activates acid hydrolases, such as cathepsin K in degrading the matrix (52, 53).

Osteoclasts differentiate through the commitment of hematopoietic stem cells to the myeloid lineage and are regulated by PU.1 together with micro-ophthalmia-associated transcription factors (54). Also, macrophage CSF/CSF-1R stimulates expression of RANK and leads to osteoclast precursor commitment. Furthermore, RANK Ligand (RANKL) is essential for osteoclast formation, function, and survival (52). Moreover, RANKL/RANK signaling induces the nuclear factor-κB (NF-κB) and nuclear factor of activated T cells cytoplasmic 1, which leads to osteoclast differentiation (55).

Recent studies have demonstrated that TSH reduces osteoclastogenesis by acting on their TSHR G-protein-coupled receptor (43, 56, 57). In animal studies, mice which lack the TSHR exhibited osteoporosis because of enhanced osteoclast formation (43). TNFα, which is a member of the tumor necrosis family, is a well-established signal that increases osteoclasts (58). The receptor activator for NFκB ligand (RANKL) stimulates endogenous TNFα expression and it is necessary for osteoclast formation. Additionally, RANKL and a mixture with IL1 and TNFα increase osteoclastogenesis (59). Moreover, we showed that the TSHR null mice exhibit an elevated TNFα expression in osteoclast progenitors. The fact that these mice develop osteoporosis (43) suggests that TNFα overproduction may play a major role in the development of this condition since TSH has been shown to directly downregulate TNFα transcription induced by IL1 or RANKL treatments (59).

## TSH Effects on Osteocytes

In contrast to osteoblasts and osteoclasts, osteocytes make up 90–95% of bone cells and are embedded in bone matrix for decades. Osteocytes have been increasingly recognized as the major orchestrator of bone activity, particularly considering the fact that they secrete a 190-amino-acid glycoprotein, which decreases bone formation by inhibiting terminal osteoblast differentiation while promoting apoptosis. These cells also regulate osteoblast physiology by controlling osteoblast and osteoclast activity during bone remodeling. Terminally differentiated osteoblasts are widely described as mature osteocytes.

However, it is poorly understood how osteoblast becomes embedded in bone matrix to begin a new life in the capacity as an osteocyte and also the molecular and genetic mechanisms, which regulate the differentiation and maturation of the osteocyte are also poorly understood (60).

Osteocytes takes up residence in lacunae within mineralized matrix and protrude their dendritic processes through the canaliculi to form a network, which connects with cells on the bone surface and to blood vessels (61).

Localized conditions such as mechanical stresses and microdamage stimulate osteocytes to release cytokines, chemotactic signals, or to induce apoptosis. An, increase in mechanical stress stimulates local bone formation through osteoblast activity, whereas reduced microdamage results in bone resorption induced by osteoclast activity (60, 62, 63). These mechanosensor capabilities of osteocytes allow them to control bone remodeling through their regulation of osteoclasts and osteoblasts *via* the RANKL/RANK pathway and modulation of Wnt signaling (60, 64). The effects of TSH on osteocytes have not been studied.

## Skeletal Consequences in the TSHR Knockout Mouse

**Figure 4:**
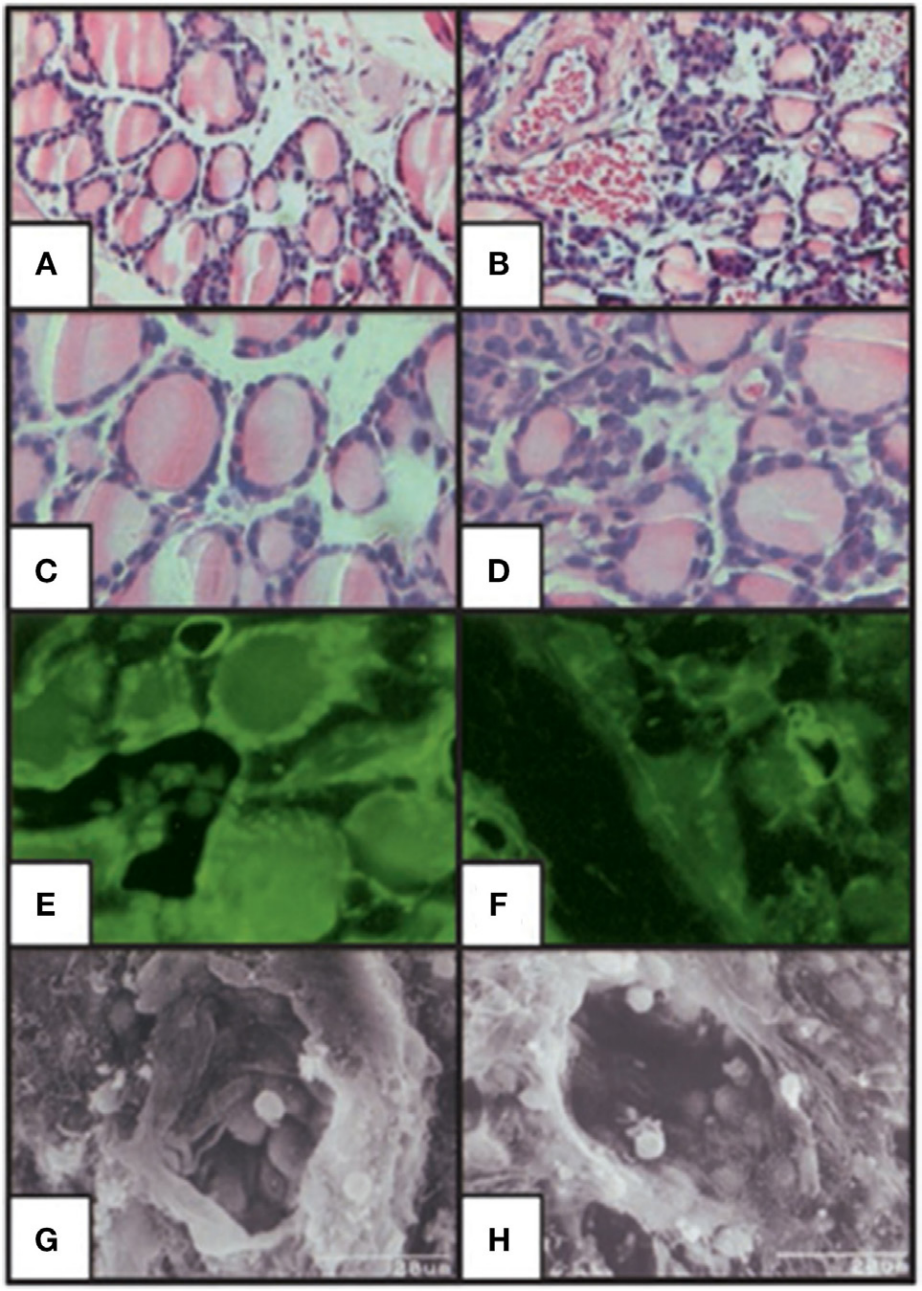
Histologic staining of the thyroid-stimulating hormone receptor (TSHR)-KO mouse thyroid gland. Hematoxylin/eosin stained sections of WT **(A,C)** and TSHR-KO **(B,D)** thyroids. Fluorescent imaging of heterozygous **(E)** and TSHR-KO **(F)** green fluorescent protein reporter gene expression in the thyroid. Scanning electron micrographs for WT **(G)** and TSHR-KO **(H)** thyroid follicles [magnification: **(A,B)** 100; **(C–F)** 400; **(G,H)** 1,500]. Note the small but present thyroid cells in the TSHR-KO mouse, which has been used in a number of studies elucidating thyroid-stimulating hormone actions on bone. Copyright (2002) National Academy of Sciences, U.S.A (71).

The use of animal models in the study of TSH effects on bone has provided important fundamental advances. Animal models of hypothyroid mice such as the Snell Dwarf mouse (65), the cog mouse (66), and the hyt/hyt mouse (67, 68) have all retained the TSHR expression and ligand-independent constitutive signals transmitted by the TSHR (69). In contrast, the generation of the TSHR-KO mouse, brought a novel way of studying TSH signaling and this implicated the TSHR in bone biology (11, 43, 70). In this mouse, exon-1 of the TSHR gene was replaced with a green fluorescent protein (GFP) cassette. The heterozygotes, haplo-insufficient in the TSHR, were euthyroid and exhibit normal growth and normal thyroid hormone and TSH levels. By contrast, the homozygotes (TSHR-KO mice) showed runted growth, low thyroid hormone levels, and very high TSH levels and required thyroid hormone replacement for normal growth and survival. Nevertheless, these mice had a smaller thyroid gland in the correct position. An examination of the TSHR-KO thyroid follicles (Figure 4) showed GFP expression in the heterozygote and homozygote thyroid follicles indicating that the TSHR had been deleted but the thyroid follicles, while appearing normal in the heterozygous, were few and small in the homozygous and their pattern was disorganized. Hence, the TSHR-KO mice showed congenital hypothyroidism with undetectable thyroid hormones and a rise in serum TSH.

Untreated TSHR-KO mice were found to have a low bone mineral density (BMD), increase bone formation, and resorption. However, even when these mice were given thyroid hormone replacement, they displayed a reduction in BMD and reduced calvarial thickness (43). Heterozygotes showed a smaller reduction in BMD, affecting only some parts of the skeleton. There were no change in calvarial thickness, and no difference in bone resorption or formation. These data indicated that TSH signaling must suppress bone loss and TSH was, therefore, proposed as an activator of bone formation and inhibitor of bone resorption (43). Because the TSHR-KO mice are only thyroid supplemented from weaning (around 21 days of age) (43), they do remain severely hypothyroid during a critical time of skeletal development but clearly are unable to catch up.

## TSH Effects on Normal Rodent Skeleton

The osteoporosis due to TSHR deficiency in the TSHR/KO mouse is of the high-turnover variety. Further, when TSH was intermittently administered into ovariectomized rats, it displayed a robust *in vivo* antiresorptive action (46, 72). TSH increased bone volume, trabecular number, trabecular thickness, and decreased trabecular separation (46). TSH also decreased osteoclast numbers in these rats (46) suggesting that TSH treatment is capable of restoring ovariectomy-induced bone loss and bone strength (72). The inhibitory action of TSH on osteoclast even persisted after therapy halted (72). This lasting antiresorptive action of TSH was mimicked in cells that genetically overexpressed a constitutively active ligand-independent TSHR (73). Additionally, due to a loss of function in congenital mutant TSHR congenital hypothyroid mice, osteoclast differentiation is activated, thus confirming that TSHR signaling has a pivotal role in the regulation of bone remodeling (72).

## TSH Effects on the Human Skeleton

As discussed earlier, new lines of evidence have shown the influence of pituitary hormones on the skeleton (43, 72, 74, 75). For example, suppressed hyperthyroid levels of TSH are well known to correlate with low BMD (76), especially in postmenopausal women, and even low normal TSH levels show the same relationship in the elderly (77) and an increased risk of hip fractures in euthyroid women (77). These studies also show that duration of TSH suppression was also a predictor of major osteoporotic fractures. However, others (78) could not distinguish the separate pharmacological effects of thyroid hormones and TSH on bone turnover, although TSH was correlated inversely with markers indicative of bone turnover and is unrelated to thyroid hormones.

For humans, few large data sets exist on the physiologic effects of TSH on bone in. However, recombinant human TSH administration regulated C-telopeptides type-1 collagen levels and alkaline phosphatase with no effect on levels of osteoprotegerin (79) or on the receptor activator of nuclear factor-κB ligand levels (80).

## T3 Effects on the Skeleton

**Figure 5:**
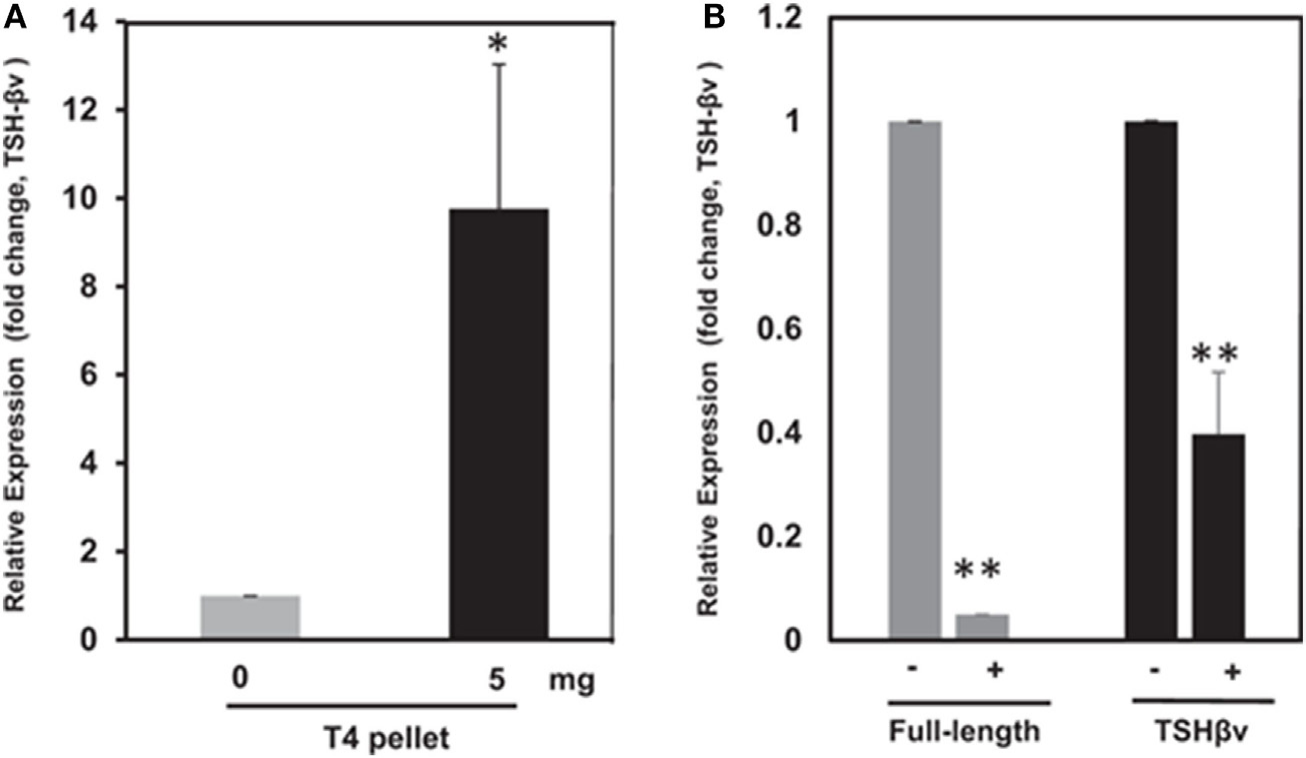
**(A)** Thyroid hormone regulation of mouse thyroid-stimulating hormone (TSH)-βv in bone marrow cells. Here, bone marrow cells from mouse WT mice subcutaneously treated with T4 hormone pellets for 21 days showed greatly increased TSH-βv gene expression. Copyright (2016) Endocrinology and reproduced with permission from Oxford University Press (3). **(B)** Thyroid hormone regulation of mouse TSH-βv in the pituitary. The pituitary tissue from WT mice administered subcutaneous T4 pellets for 21 days showed suppression of both wild-type TSH-β and TSH-βv. This is in contrast to the bone marrow cells shown in **(B)**. Copyright (2016) Endocrinology and reproduced with permission from Oxford University Press (3).

Thyroid hormone levels have a major influence on bone homeostasis (81), and this has been well reviewed elsewhere (5). Investigators have focused on the direct effects of the active thyroid hormone (T3), on bone cells, *via* the thyroid hormone receptor family that induces transcription in a ligand-dependent manner (82). Osteoblasts express thyroid hormone receptors (TRs) (TRα1, TRα2, and TRβ1) and respond to T3 with increased proliferation and expression of lineage-specific markers such as alkaline phosphatase, osteocalcin, and collagen. Interestingly, although osteoclasts have TRs, their response to T3 appears to be mediated mostly by osteoblasts since T3 induces osteoblasts to express RANKL, the key osteoclastogenic cytokine. Additionally, mice lacking the known active isoforms of TRs have retarded bone growth and maturation, but do not manifest increased BMD, as would be predicted if T3 was an important stimulus of bone resorption in the euthyroid state (83). Further, T4, the prohormone of T3, suppressed pituitary TSH release but enhanced bone marrow TSHβv expression (3) (Figures 5A,B) indicating an attempt at osteoprotection. Hence, our observation of enhanced bone loss induced by T4 when the TSHR is absent fits with these correlative data (11).

## Summary and Conclusion

**Figure 6:**
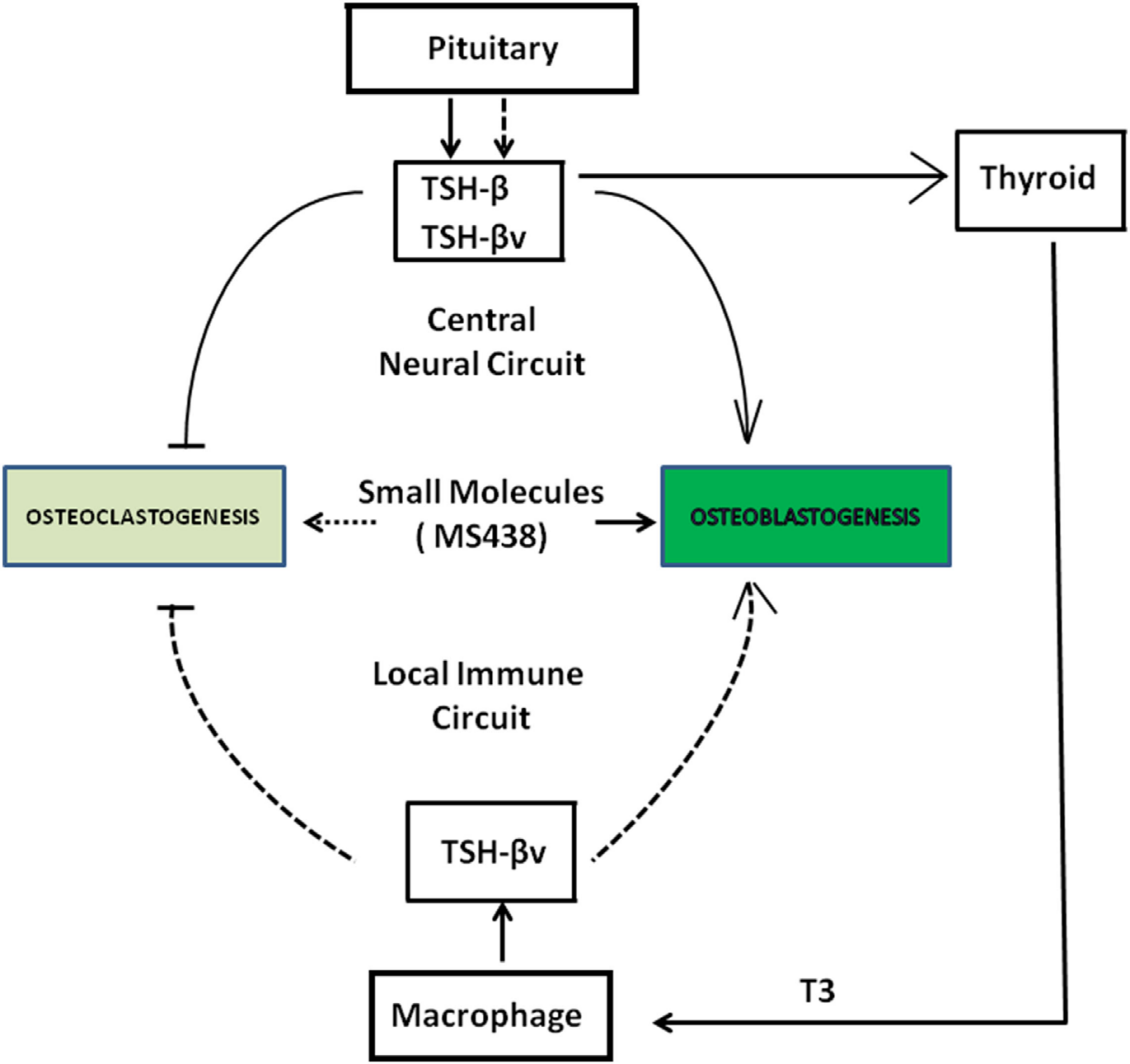
Schematic showing a collaborative effort among pituitary, thyroid, and macrophages in the local bone marrow microenvironment in regulating osteoclast and osteoblast activity through the release of thyroid-stimulating hormone (TSH)-β and TSH-βv. SM 438 is a small molecule agonist at the thyroid-stimulating hormone receptor. Solid dark arrows indicate modulatory effects of TSH-β, TSH-βv, and SM 438 on bone cells and the broken arrows indicate that such effects still need to be mapped.

Thyroid-stimulating hormone, TSH-β, and TSH-βv are produced through central neural circuits in the pituitary thyrotrophs and are negatively regulated by T3 produced by the thyroid gland. However, in the local peripheral immune circuit, only TSH-βv is produced by bone marrow macrophages and appears to be positively regulated by T3. It has been shown that intact TSH exerts anabolic and osteoprotective effects on bone by stimulating osteoblast differentiation and by inhibiting osteoclast formation and survival. Since the TSHR is widely distributed in bone cells, the production of TSH-βv by macrophages argues for a local TSH-TSHR circuit regulating bone physiology. Evidence for the importance of such influences is shown by the greater T4-induced bone loss in the absence of TSHR signaling (Figure 6).

## Author Contributions

RB, RL, MZ, and TF contributed to the design, figures, and writing of this manuscript.

## Conflict of Interest Statement

TD is on the Board of Kronus Inc., Star, ID, USA. The remaining authors have nothing to disclose. The reviewer IR and handling editor declared their shared affiliation.
